# ZEB1 Is a Transcription Factor That Is Prognostic and Predictive in Diffuse Gliomas

**DOI:** 10.3389/fneur.2018.01199

**Published:** 2019-01-17

**Authors:** Lincoln A. Edwards, Sungjin Kim, Mecca Madany, Miriam Nuno, Tom Thomas, Aiguo Li, Dror Berel, Bong-Sup Lee, Minzhi Liu, Keith L. Black, Xuemo Fan, Wei Zhang, John S. Yu

**Affiliations:** ^1^Neurosurgery Department, Cedars-Sinai Medical Center, Los Angeles, CA, United States; ^2^Biostatistics and Bioinformatics Research Center, Cedars-Sinai Medical Center, Los Angeles, CA, United States; ^3^Neuro-Oncology Branch, National Cancer Institute, National Institutes of Health, Bethesda, MD, United States; ^4^Pathology and Laboratory Medicine Department, Cedars-Sinai Medical Center, Los Angeles, CA, United States

**Keywords:** ZEB1, copy number, decision curve analysis, diffuse gliomas, glioma stem cells (GSCs)

## Abstract

**Objective:** To address the unmet medical need to better prognosticate patients with diffuse gliomas and to predict responses to chemotherapy regimens.

**Methods:** ZEB1 alterations were retrospectively identified from a cohort of 1,160 diffuse glioma patients. Epigenome-wide association scans (EWAS) were performed on available data. We determined the utility of ZEB1 as a prognostic indicator of patient survival in diffuse gliomas and assessed the value of ZEB1 to predict the efficacy of treating diffuse glioma patients with procarbazine, CCNU, and vincristine along with radiation at diagnosis. Decision curve analysis (DCA) was used to determine if ZEB1 added benefit to clinical decision-making over and above conventional methods.

**Results:** Fifteen percent of diffuse glioma patients had a *ZEB1* deletion. *ZEB1* deletion was associated with poor overall survival (OS) with and without adjustment for age and tumor grade (adjusted HR: 4.25; 95% CI: 2.35 to 7.66; *P* < 0.001). Decision curve analysis confirmed that ZEB1 status with or without IDH1 was more beneficial to clinical decision making than conventional information such as age and tumor grade. We showed that ZEB1 regulates TERT expression, and patients with ZEB1 deletions likely subsume patients with mutant TERT expression in diffuse gliomas. *ZEB1* influenced clinical decision making to initiate procarbazine, CCNU, and vincristine treatment.

**Conclusion:** We demonstrate the prognostic value of ZEB1 in diffuse glioma patients. We further determine ZEB1 to be a vital and influential molecular marker in clinical decisions that exceed conventional methods regarding whether to treat or not treat patients with diffuse glioma.

## Introduction

Diffuse gliomas, comprised of WHO grade II and grade III astrocytomas, oligodendrogliomas, and diffuse gliomas with ambiguous histology (formerly known as oligo-astrocytomas) are infiltrative malignant tumors of the central nervous system (1). In contrast to glioblastomas, diffuse gliomas have a longer patient survival but ultimately progress to secondary glioblastomas. Although histology has been the cornerstone of the classification of gliomas and treatment decision making, the variation that accompanies histologic classification (due to intraobserver and interobserver variability) does not satisfactorily predict clinical outcomes (2, 3). Next generation sequencing has led to the identification of genes that acquire somatic mutations such as *IDH1, TERT*, and *TP53* which contribute to diffuse gliomas. Already genetic classification is more readily being incorporated into prognostic classification of these tumors (4, 5). Treatment efforts have been confounded due to the lack of actionable information based on newly acquired genetic information. We recently determined that *ZEB1* (Zinc finger homeobox gene), an epithelial to mesenchymal transition (EMT) transcription factor that promotes invasion and metastasis in carcinomas, was deleted in approximately half of glioblastomas (6). ZEB1 is part of a family of transcription factors (ZEB1 and ZEB2 in vertebrates) that bind to canonical DNA binding motifs with the ability to act as both an activator (6, 7) or a repressor (8, 9). Even more curious than ZEB1 being described to have opposing functions, there has been opposing evidence with regard to its functions in cancer and cancer stem cell like characteristics (10–15). At the forefront of the ZEB1 dichotomy is the role of ZEB1 in glioblastomas. For example, Dai et al. indicated that the histone demethylase KDM5A could suppress ZEB1 activity leading to decreased invasion with an implication that patient survival is shorter with increased ZEB1 expression (16). Similarly, long non-coding RNAs of antisense ZEB1 (ZEB1-AS1) which upregulate ZEB1 was shown to correlate with shorter patient survival (17). Micro-RNAs such as mIR-200 and miR-141 were also shown to inhibit ZEB1 resulting in decreased glioma growth suggesting that ZEB1 expression is the resulting cause of glioma progression (18). It is important to note that all of these studies were performed using conventional glioma cell lines and not patient derived glioma stem cells, which have been shown to be genotypically and phenotypically closer to glioblastomas found within patients (19–22). Using glioblastoma stem cells Siebzehnrubl et al. found that increased ZEB1 was associated with glioblastoma initiation, invasion and chemoresistance (6). Adding to the complexity of ZEB1 we previously demonstrated that the loss of *ZEB1* imparts “stemness” to cancer stem cells derived from glioblastoma to prevent differentiation and induce self-renewal. This property was executed by Leukemia Inhibitory Factor (23) whose expression is inhibited by ZEB1. To determine how *ZEB1* deletion (*ZEB1*^del^) and expression might impact patient prognosis, glioma classification, and subsequent clinical decision making for therapy, we performed multi-dimensional analysis on adult patients from over 381 brain cancer genomes of diffuse gliomas for copy number analysis, and over 770 gliomas for mRNA expression analysis. We further analyzed diffuse glioma patient samples for DNA methylation and epigenome-wide association scans (EWAS) to determine epigenetic variation, which may account for changes in ZEB1 expression that could also contribute to poor patient outcomes. In addition, we utilized ZEB1 in decision curve analysis (DCA) to determine if this molecular marker provides a benefit in clinical decision making over the standard evaluation of utilizing age and tumor grade. Here, we describe the use of the molecular marker *ZEB1* in prognostication and clinical decision making for patients with diffuse gliomas.

## Materials and Methods

### Search and Selection Criteria

Thousand one hundred and sixty samples consisting of diffuse gliomas were compiled and investigated. Copy number, DNA methylation and gene expression analysis, and all clinical data were collected from datasets. These datasets included The Cancer Genome Atlas (TCGA), cBioportal (24), ArrayMap (25), Gene Expression Omnibus (GEO) (26), Methylation (27), and Nexus Biodiscovery. TCGA numbers, matched with mutation status, patient age, and time of death were identified and overlapping patients were removed.

These authors (LE, SK, MN, AL, DB.) accessed databases and datasets; all data were screened independently by each author. To be eligible, datasets had to meet the following criteria: grades II and III gliomas, with either ZEB1 expression or *ZEB1* copy number data and *IDH1* copy number or expression data. The incorporation of patient age and histology utilized TCGA datasets or data from published literature. We included cohorts whether prospectively or retrospectively defined and studies that pooled datasets. All data was consolidated. Discrepancies in cohorts, datasets or selection criteria were resolved by discussion between the reviewers until an agreement was reached.

### Data Extraction

Data extraction consisted of collecting information regarding the (1) tumor grade (2) histology (3) genes and/or expression, (4) DNA methylation or copy number information (5) treatment, and (6) outcomes such as survival, whenever possible. Gene expression was dichotomized at the median to determine high and low expression of mRNA. Copy number was determined either by previous analysis that was deposited in TCGA, ArrayMap, or Nexus biodiscovery.

### Luciferase Reporter Assays

In order to measure the transcriptional activity of TERT in 293T cells (1 × 10^5^ cells per transfection, three replicates per condition), 293T cells were transiently transfected with a TERT luciferase promoter reporter plasmid (1 μg; Switchgear genomics) and co-transfected with or without a ZEB1 expression construct (1 or 3 μg; Origene) using a Lonza Nucleofector machine. The cells were cultured for 48 h, harvested and assayed for luciferase activity with the use of a GloMax 20/20 luminometer (Promega) in accordance with the manufacturer's instructions. Luciferase activity was expressed relative to that of cells transfected with a control plasmid containing a minimal luciferase promoter.

### Decision Curve Analysis (DCA)

The risk to harm ratio is an important concept when deciding whether to initiate a treatment. The implementation of such risk to harm predictions to decide the usefulness of a certain therapy can be achieved using DCA. The clinical usefulness of the prediction models was evaluated by DCA deriving the net benefit of the models across a range of thresholds for mortality at 2-years, with visualization in a decision curve. The principle is that the relative harms of false positives (e.g., unnecessary treatment) and false negatives (e.g., missed death) can be expressed in terms of a probability threshold, and it can help to identify the range of threshold probabilities where a model is of value and the magnitude of benefit is shown. This will allow us to make decisions about whether to use a model or which model is more informative to use (28). The R code for DCA can be found at http://www.decisioncurveanalysis.orgalong with tutorials on using the code.

### Statistical Analyses

Data were presented as frequency (percentage, %) for categorical variables and median (interquartile range, IQR) for continuous variables. The primary outcome was overall survival (OS) calculated from diagnosis to the date of death or censor at last follow-up. Univariate associations between variables were examined with Wilcoxon rank-sum test, Kruskal-Wallis test, chi-square test, or Fisher's exact test, where appropriate. Survival functions were estimated by the Kaplan-Meier method and the log-rank test was used to assess the difference in OS stratified by *ZEB1* or *IDH1/ZEB* (29). Univariate and multivariable survival analyses were carried out using a Cox proportional hazards model (30). The proportional hazards assumption was assessed graphically and analytically with scaled Schoenfeld residuals (31). Variable selection was carried out as outlined by Collett (32) and the possibility of *multicollinearity* was assessed by tolerance and the variance inflation factor. The Benjamini and Hochberg method was used to control false-discovery rate for multiple comparisons (33). The prediction models were assessed in terms of measures of discrimination and calibration (34). The models' discriminative ability was measured using c-statistics (35) and the calibration of the model predictions was graphically assessed with predicted vs. observed probability based on the loess algorithm. Internal validation for the models was performed by estimating and correcting possible overfitting in the model performance estimates using the bootstrap method with 1,000 replicates (36). All statistical analyses were performed using SAS 9.4 (SAS Institute, Inc., Cary, North Carolina) and R package version 3.5.0 (*rms* and *survival* libraries; The R Foundation for Statistical Computing) with two-sided tests and a significance level of 0.05.

## Results

### Patient Characteristics

An initial 334 diffuse glioma patients were assessed for *ZEB1* copy number along with age, histological type and tumor grade. Sixty three diffuse glioma (DG) patients had a *ZEB1*^del^ (19%) with the majority of diffuse glioma patients having an oligodendroglioma of grade II or III (40%), followed by astrocytoma (34%), and the smallest group consisting of diffuse glioma with ambiguous histology with grades of II or III (26%). Associations of *ZEB1* genotypes with *IDH1* genotypes, histology, grade, and age are noted in Table 1.

**Table 1:** Univariate and multivariable overall survival analyses with ZEB1 alone and IDH/ZEB1 in addition to clinical variables in patients with grade II/III gliomas.

**Variable**	**Univariate**			**Multivariable**					
				**With ZEB1^a^**		**With IDH/ZEB1^b^**		**Clinical Variables^c^**	
	***N***	**Hazard ratio (95% CI)**	***P*-value**	**Hazard ratio (95% CI)**	***P*-value**	**Hazard ratio (95% CI)**	***P*-value**	**Hazard ratio (95% CI)**	***P*-value**
Age at diagnosis	334	1.07 (1.05–1.10)	<0.001	1.06 (1.04–1.09)	<0.001	1.06 (1.04–1.08)	<0.001	1.07 (1.05–1.09)	<0.001
Histologic type			0.073^*^						
Astrocytoma	112	1.88 (1.08–3.30)	0.027	^†^		^†^		Not included	
Ambiguous histology^**^	87	1.16 (0.57–2.35)	0.677						
Oligodendrogliomas	135	1 (Reference)							
**GRADE**									
II	153	0.32 (0.18–0.56)	<0.001	0.55 (0.30–1.01)	0.056	0.56 (0.30–1.03)	0.060	0.38 (0.21–0.67)	<0.001
III	181	1 (Reference)		1 (Reference)		1 (Reference)		1 (Reference)	
**1p/19q CO-DELETION STATUS**									
True	76	0.51 (0.26–0.99)	0.048	^†^		^†^		Not included	
False	138	1 (Reference)							
**ZEB1**									
CN deletion	63	7.20 (4.24–12.24)	<0.001	4.25 (2.35–7.66)	<0.001	Not included		Not included	
Wildtype	271	1 (Reference)		1 (Reference)					
IDH/ZEB1			<0.001^*^				<.001^*^		
IDHmut-ZEB1wt	257	0.07 (0.04–0.13)	<0.001	Not included		0.13 (0.06–0.27)	<0.001	Not included	
IDHwt-ZEB1wt	14	0.16 (0.05–0.48)	0.001			0.31 (0.10–0.97)	0.044		
IDHmut-ZEB1del	8	0.18 (0.06–0.57)	0.004			0.26 (0.08–0.85)	0.026		
IDHwt-ZEB1del	55	1 (Reference)				1 (Reference)			
Optimism-corrected c-statistic (95% CI)				0.832 (0.745, 0.919)		0.841 (0.754, 0.928)		0.813 (0.726, 0.900)	

* *Overall p-value for variables with more than two categories*.

** *Formerly Oligoastrocytoma*.

† *Dropped out of the final model*.

*334 observations were used in multivariable models*.

a *Multivariable model including ZEB1 as well as clinical variables*.

b *Multivariable model including IDH/ZEB1 as well as clinical variables*.

c *Base model without a predictor variable of either ZEB1 alone or IDH/ZEB1*.

### Copy Number Alterations in ZEB1

**Figure 1:**
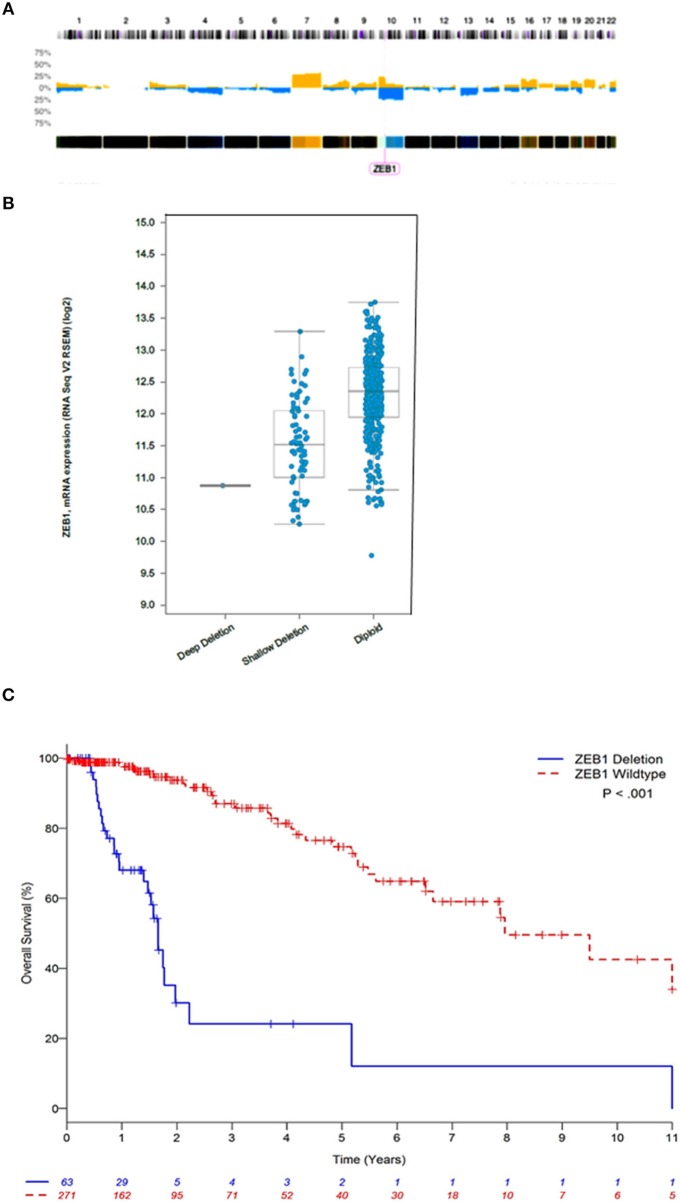
ZEB1 copy number aberrations in low grade gliomas. **(A)** Histogram of copy-number of low grade glioma patients. This plot represents an aggregate of low grade gliomas. Percentage values in Y axis corresponding to numbers of gains (yellow) and losses (blue) account for the whole dataset. **(B)** *ZEB1* deletion represented by deep deletion (homozygous), shallow deletion (heterozygous), diploid (wildtype), corresponding to the ZEB1 expression level represented on the Y axis. **(C)** Estimated Kaplan-Meier survival curves based on copy number for ZEB1 low grade gliomas patients ^**^P < 0.001. *ZEB1* deletion for low grade glioma, defined as copy number less than or equal to −0.5 (*n* = 63); wildtype (WT) defined as copy number greater than or equal to zero (*n* = 271). Two-tailed student *t*-test identified a significant difference between these two groups ^**^P < 0.001.

We interrogated 46 DG samples through whole genome copy number analysis (Figure 1A), where we identified almost universal copy number alterations (CNAs) on chromosome 10 (p11.22). This is the first report of highly variable diffuse gliomas with *ZEB1* CNAs significantly below wildtype copy number. In this initial cohort, we identified 30/46 DG samples (63%) with *ZEB1*^del^. In stratifying expression and *ZEB1* copy number the *ZEB1*^del^ were wholly heterozygous in nature and were associated with a decrease in expression of ZEB1 (Figure 1B).

### ZEB1 and Patient Survival

We observed that *ZEB1* copy number loss in DG was associated with poor OS (Table 1 and Figure 1C, Supplementary Figure 1). In multivariable analysis, *ZEB1* copy number loss remained significantly associated with poor OS after adjusting for age and grade (HR: 4.25; 95% CI: 2.35–7.66; *P* < 0.001, Table 1). Histologic type was not associated with OS in the multivariable model. To investigate the additional value of *ZEB1* copy number loss vs. wildtype on OS, the model including *ZEB1* copy number in addition to age and tumor grade was compared with the base model including age and tumor grade (Table 1). After correcting for possible over-fitting, there was a trend toward improved predictive accuracy by adding *ZEB1* copy number to the base model including age and tumor grade (change in optimism-corrected c-statistics: 0.02; 95% CI: −0.01 to 0.05) though it was not statistically significant.

**Figure 2:**
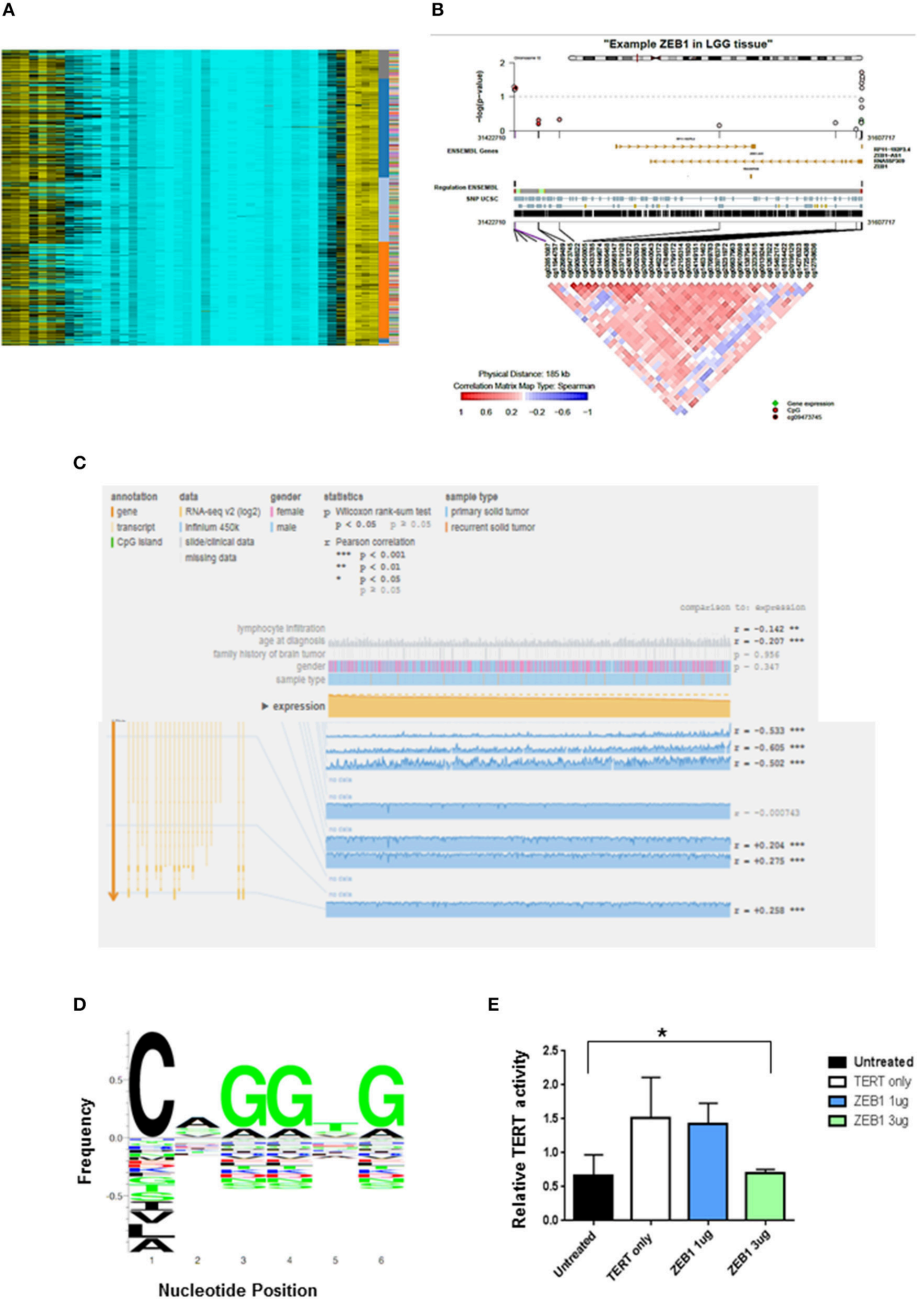
ZEB1 DNA Methylation in low grade gliomas. **(A)** Heat-map representation of an unsupervised clustering of DNA methylation profiles of 434 low grade glioma tumors. Each row represents a probe; each column represents a sample. The level of DNA methylation (beta value) is represented with a color scale methylated (yellow) and unmethylated (blue). Sample, subgroup association, and patient ID are indicated at the right. **(B)** Representative coMET plot of ZEB1 methylation in a low grade glioma patient to identify methylated CpG probe clusters. The coMET plot generates localized plots of estimated DNA methylation correlation between CpG sites (co-methylation). **(C)** Mexpress plot was used to further specify DNA methylation. A negative correlation can be identified between increased ZEB1 DNA methylation and ZEB1 expression in low grade glioma patients. **(D)** ZEB1 DNA binding domain sequence identified by TERT promoter analysis and motif enrichment. The letter height indicates the occurrence frequency which is denoted by the Y-axis. And the corresponding nucleotide at each position denoted by the X-axis. **(E)** A luciferase TERT promoter reporter was transiently transfected into 293T cells with or without transient transfection of certain ZEB1 expressing construct concentrations. The relative luciferase level indicates TERT promoter activity and is expressed on the Y-axis. TERT activity was substantially decreased with transient transfection of ZEB1. Experiments were quantified by one-way ANOVA, ^*^P < 0.05.

*ZEB1*^del^ due to copy number loss was associated with decreased expression of ZEB1 and shorter patient survival in DG. To determine other mechanisms besides copy number loss that could impact patient survival, we explored the potential impact of epigenetic silencing as a mechanism to account for decreased ZEB1 transcriptional expression. We looked at DNA methylation as a means of gene silencing. Visualization of diffuse gliomas from illumina450K Bead Chip array in a non-supervised analysis indicated DNA methylation of *ZEB1* in DG patients (Figure 2A). The pattern of methylation in DG patients suggested that methylation was occurring in clusters in genomic regions. This is consistent with hypermethylation and therefore potentially a silencing affect. To determine if in fact CpG clusters could be identified for the *ZEB1* gene we used epigenome wide association scans (EWAS). To do EWAS we used the CoMET algorithm (37). Methylated regions were identified for the *ZEB1* gene and suggested clustering around certain CpG sites, again implying the potential for the *ZEB1* gene to be silenced (circles indicating CpG methylation). A correlation matrix indicating an association of methylated probe clustering (red portion of the heatmap) also suggested hypermethylation (Figures 2B,C). The data derived indicated that as expression decreased methylation increased in these DG patients, indicating that methylation albeit in a few patients (5/567, 0.9%) was associated with a decrease in ZEB1 gene expression (Figure 2C).

### Impact of ZEB1 on TERT Expression

We have been able to show the prognostic value of *ZEB1* alone and in conjunction with *IDH1* for patient outcome (Table 1). Recent evidence has suggested that another molecular marker, telomerase reverse transcriptase (TERT), has prognostic value in DG patients (4). DG patients with a *TERT* mutation alone have a shorter OS than do DG patients with wildtype *TERT*. Interestingly, we identified within the *TERT* promoter a canonical binding site (CANNTG) for ZEB1—known to bind to Ebox motifs (Figure 2D). Using a TERT promoter-reporter in conjunction with a ZEB1 expression construct, we determined that ZEB1 binding negatively regulated TERT expression (Figure 2E). This suggests that loss of *ZEB1* due to deletion in the context of *TERT* mutation, which increases TERT expression, is a mechanism that leads to a decrease in the survival of diffuse glioma patients. However, we recognize that binding could be indirect which may influence our suspected role for ZEB1. Further analysis which is beyond the scope of this manuscript will need to be performed.

### ZEB1 in Conjunction With IDH1 and Patient Survival

In order to determine the consequence of *ZEB1*^del^ on patients with DG in relation to other known genes from unbiased genetic analyses, we analyzed *ZEB1*^del^ in conjunction with the status of IDH1, the standard molecular marker in defining DG patient prognosis. We first classified diffuse glioma patients into four categories: *IDH1* mutation (*IDH1*^R132MUT^) and *ZEB1* wildtype (*ZEB1*^WT^), *IDH1* wildtype (*IDH1*^WT^) and *ZEB1*^WT^, *IDH1*^R132MUT^ and *ZEB1*^del^, and *IDH1*^WT^ and *ZEB1*^del^, and examined their association with OS with and without adjustment for age and tumor tissue grade (Supplementary Table 1). *IDH1/ZEB1* was associated with OS (Supplementary Table 1 and Supplementary Figure 1), and it remained a significant predictor of OS after adjusting for age and grade with *IDH1*^WT^ and *ZEB1*^del^ associated with a higher risk of death as compared to others while *IDH1*^R132MUT^ with *ZEB1*^WT^ was associated with an improved OS (Table 1). By adding *IDH1/ZEB1* to the model with age and grade, predictive accuracy improved by 0.03 (95% CI: −0.00 to 0.06) after correction for potential over-fitting.

### Decision Curve Analysis

To determine whether *ZEB1* and *IDH1*^R132MUT^ status would be predictive of benefit from a therapy we explored the utility of *IDH1/ZEB1* status in determining the therapeutic benefit of using procarbazine, CCNU, and vincristine through a DCA.

**Figure 3:**
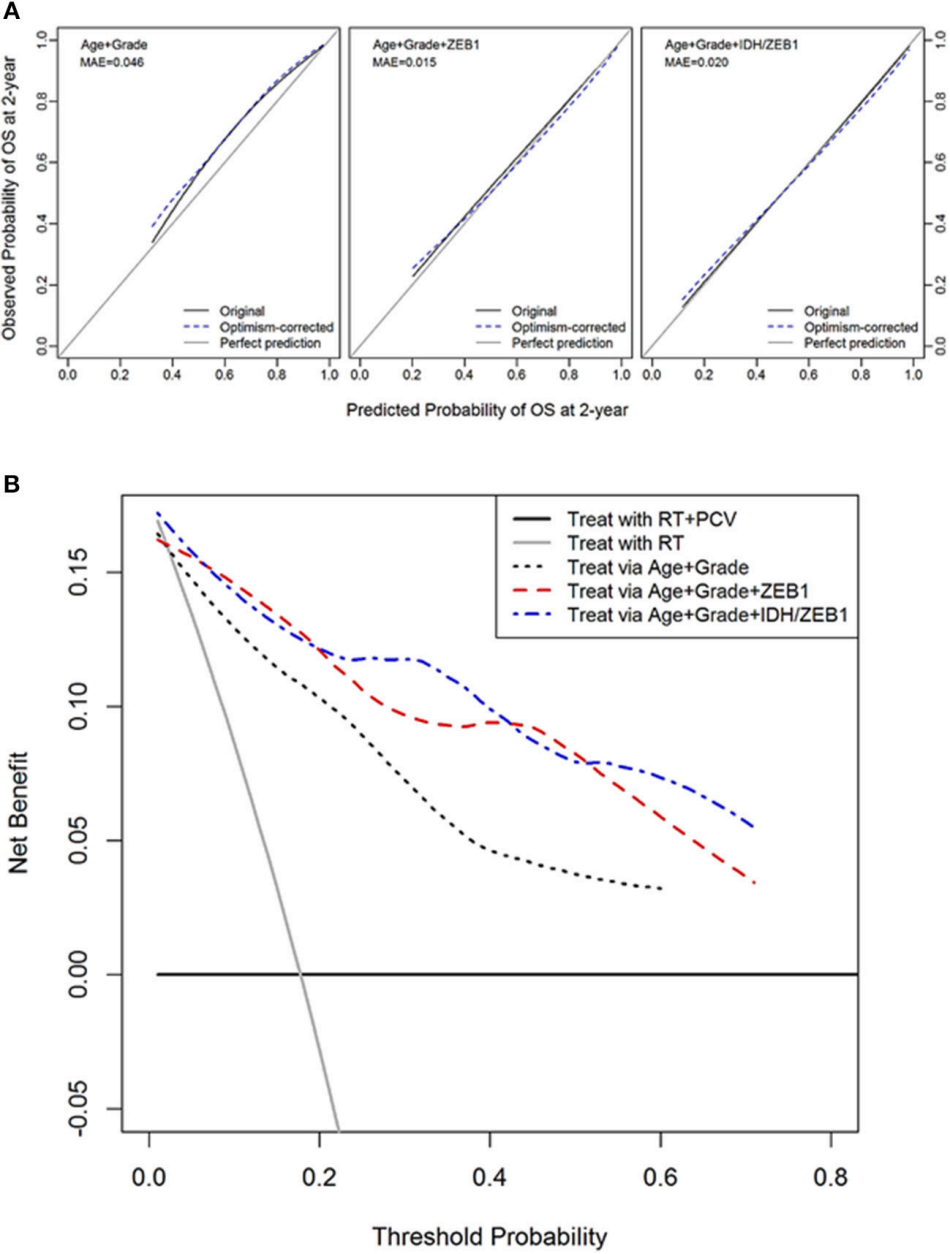
Decision curve analysis of ZEB1 and ZEB1/IDH1. **(A)** Calibration plots for 2-year overall survival with models with and without ZEB1 or IDH/ZEB1 in patients with grade II/III gliomas. The 45° line is a reference line indicating a perfect prediction; the black curve indicates the performance of the model; and the blue dotted curve indicates optimism-corrected estimates by bootstrapping with 1,000 replicates. **(B)** Decision curve analysis for 2-year mortality with the models with and without ZEB1 or IDH/ZEB1 in patients with grade II/III gliomas. Decision curve for the model without ZEB1/IDH1, with ZEB1, and with ZEB1/IDH1 to predict treatment within 2 years of diagnosis with age and/or grade. The small gray line indicates the net benefit for “treat all,” while the horizontal line indicates “treat none.” These 2 lines serve as a reference for the lines for the net benefit of models with or without the molecular markers ZEB1/IDH1. We see that the predictions get better with use of the molecular markers ZEB1 alone or with ZEB1 and IDH1 together with the conventional determinants of age and grade. RT, radiation therapy; PCV, procarbazine, CCNU, and vincristine.

Decision curve analysis was performed to compute the net benefit of decisions to initiate chemotherapy with procarbazine, CCNU, and vincristine (PCV) based on the ZEB1 molecular marker along with age and tumor grade. The desirable outcome, based on RTOG 9802 reports was an increase in survival in those patients that received PCV in addition to radiation as compared to control patients who only received radiation (38, 39). This randomized phase III trial demonstrated the benefit of adding PCV to upfront radiation for the treatment of grade II diffuse glioma. However, to derive a benefit, patients had to survive for at least 2 years as first noted by Shaw et al. (38) and reiterated by Bruckner et al. (39). van den Bent et al. also noted in grade III oligodendroglioma patients that the benefit to survival was seen after 2 years on this chemotherapy regimen (40). The benefit was defined as a survival benefit as a result of PCV chemotherapy while harm was defined as subjecting patients to the risks of PCV chemotherapy including neuropathy and hematotoxicity when they are not likely to survive at least 2 years in order to benefit from the treatment. The DCA creates a curve of net benefit as a function of threshold probability of survival to 2 years at which the potential benefit and harm of PCV treatment is considered to be equivalent. In DG, the molecular marker ZEB1 with or without *IDH1* provided a reasonable potential clinical benefit to choose patients that would most likely benefit from therapy vs. those that would not (since they would not survive at least 2 years) relative to the standard predictors of patient age and tumor grade (Figures 3A,B).

## Discussion

The complexity of ZEB1 in being both a transcription factor that can activate and paradoxically repress particular genes has led to conflicting results within the field. The data from Siebzehnrubl et al. (6) has shown using glioma stem cells that an increase in ZEB1 results in glioma initiation and correlated with shorter glioblastoma patient survival (6). In contrast, we have shown that it is the loss of ZEB1 that is responsible for these same effects. Two things are of note in the studies. Our patient population analyzing glioblastomas was significantly larger with >200 patients compared to >20 patients with ZEB1 loss or decreased expression (6, 15). Siebzehnrubl et al. (6) also reports that immunohistochemical staining of patient glioblastomas for ZEB1 resulted in ~50% of glioblastoma patients being negative for ZEB1 staining, for which they provide no explanation. It is our opinion that this is consistent with our account of ZEB1 deletion of which we see a frequency of 50% loss of ZEB1 in glioblastoma patients which corresponds to a shorter patient survival. Other reports, in lung (41) for example, can certainly be context, cell and tumor dependent in addition to using conventional cancer cell lines and not cancer stem cells. Yet we still appreciate the complexity of ZEB1 which even conflicts with our own data, as we have previously shown that ZEB1 is involved in the invasive process in glioma stem cells (8). Consistent with this account Kahlert et al. has shown similar findings (42). A possible reconciliation of these findings is that there may be two populations of GSCs containing both high and low expression and/or copy numbers of ZEB1. This would be consistent with the heterogeneity of some brain tumors. Such populations of high and low expressing GSCs could be exacerbated by the sampling of tumor tissue for high throughput analysis such as copy number, gene expression, and methylation analyses.

We have demonstrated that ZEB1 loss is associated with a decrease in patient survival. Loss of *ZEB1* by copy number loss, and to a lessor extent, *ZEB1* DNA methylation can result in the decreased expression of ZEB1. Loss of copy number via heterozygous deletions results in shorter patient survival for patients with DG and *ZEB1*^del^. Furthermore, we also observed that decreased expression of ZEB1 in DG patients can also result in shorter patient survival. One mechanism by which ZEB1 may undergo decreased expression is via increased DNA methylation. We have identified in some DG patients a strong correlation between increased *ZEB1* DNA methylation and decreased *ZEB1* expression. Increased ZEB1 expression has been observed previously in IDH1-mutant diffuse glioma patients (43). This increase in expression was associated with increased survival. Our demonstration of *ZEB1*^del^ as well as methylation as a secondary feature leading to the decrease in ZEB1 expression suggests that rather than *IDH1*^R132MUT^ driving up-regulation of ZEB1, *ZEB1* heterozygous deletion associates with *IDH1*^WT^. Our previous demonstration that ZEB1 is a negative regulator of LIF, a gene that induces glioma stem cell propagation, suggests that the deletion of ZEB1 may impart more stemness to tumors that lose ZEB1 expression.

The potential to utilize ZEB1 as a prognostic marker for diffuse glioma patients seems clear. The loss of the *ZEB1* gene led to a greater increase in the hazard ratio than the increase in hazard ratio from a mutated *IDH1* gene to a wildtype *IDH1* gene. This finding as well as the frequent loss of ZEB1 suggests that ZEB1 may have a significant impact on survival similar to *IDH1*. Whereas, a mutation in *IDH1* appears to be associated with an increase in survival, the heterologous deletion of *ZEB1* appears to be associated with a significant decrease in survival. The respective explanations for these outcomes are that *ZEB1* loss results in the increase of stemness of the glioblastoma whereas the *IDH1*^R132MUT^ results in inactivation of *IDH1* from the use of isocitrate as a metabolic substrate. Since the *IDH1* and *IDH2* mutations appear to only occur in DG prior to dedifferentiation into secondary gliomas and because these deletions occur in both oligodendrocytic and astrocytic lineages, the probability that the *IDH1*^R132MUT^ occurs in the stem cell from which both cell types arise has been raised (44). The loss of *ZEB1* increases stemness of gliomas and this mutation presumably occurred at the stem cell level. The co-occurrence of these mutations in the oligo-astro progenitor cell is a compelling possibility. *ZEB1* loss occurs frequently with *IDH1*^WT^ and leads to the worst prognosis. *ZEB1*^WT^ is associated most frequently with *IDH1*^R132MUT^ and leads to the best prognosis. The other permutations (*ZEB1*^del^/ *IDH1*^R132MUT^ and *ZEB1*^WT^/*IDH1*^WT^) are associated with intermediate prognosis suggesting that the mutations of either genes temper the outcome of the other gene.

We found ZEB1 binding sites within the TERT promoter that negatively regulate TERT expression. This suggests that loss of *ZEB1* due to deletion in the context of *TERT* mutation may be a mechanism that leads to a decrease in the survival of DG patients. The association of *ZEB1*^del^ with *IDH1*^WT^ leads to a decrease in *ZEB1* which allows for constitutive mutant *TERT* activation in DG patients. Conversely, *ZEB1*^WT^ coexisting in the context of *IDH1*^R132MUT^ may inhibit TERT expression in *TERT* mutated patients. This finding has implications for the importance of ZEB1's role as a transcription factor that impacts both stemness as well as TERT expression. In addition, the association of *TERT* mutations' positive survival impact on *IDH1* mutated tumors but not in *IDH1*^WT^ tumors may be due to the higher frequency of *ZEB1*^del^ in *IDH1*^WT^ tumors and the decrease of ZEB1's inhibition of TERT expression. The role of 1p/19q and IDH add yet another layer of complexity with respect to patient outcome as patients with primarily *IDH1*^R132MUT^ with 1p/19q co-deletion have been shown to have a more favorable prognosis in low grade gliomas (oligodendrogliomas) with a median survival of 8 years compared to *IDH1*^R132MUT^ with no 1p/19q co-deletion (astrocytoma) which had a median survival of 6.4 years (45). These findings are of interest to us, particularly how 1p/19q could be incorporated into our findings. We have seen that OS is increased with the addition of 1p/19q in our univariate analysis but was not significantly associated with OS in multivariate analysis (Supplementary Table 1 and Supplementary Figure 1).

Decision curve analysis was used to determine whether the use of the molecular marker ZEB1 allowed for a more informed decision whether treatment with procarbazine, CCNU, and vincristine is advisable. The addition of ZEB1 to age and tumor grade improved the ability to decide when to use procarbazine, CCNU, and vincristine in the treatment of patients with DG. Randomized trials demonstrated the efficacy of this regimen in grade II and grade III tumors and that a benefit was derived from the regimen if patients survived at least 2 years (at which point the survival curves diverge toward benefit in the treated group). Therefore, the assumption of this model was not only that patients needed to survive at least 2 years in order to benefit from the treatment, but that those patients that derived a benefit would have survived 2 years without treatment. The crossing of the survival curves after 2 years in both the grade II trial (39) and the grade III trial (40) would suggest that this is an acceptable assumption. This exercise of implementing the DCA using large trial data may be used to model the benefits and risks of therapies using biomarkers that were not used during the trial. Given the limited trial data we have examined we believe this to be a good beginning to future studies examining if other trial data or the development of a trial where we can further test the veracity of incorporating ZEB1 will add a benefit. In addition, with the advent of IDH status in gliomas it would be interesting to see how incorporation of both IDH and ZEB1 would benefit patients. The benefit of using ZEB1 alone or with IDH1 was alternately superior in different parts of the curve. Therefore, both ZEB1 alone or in combination with IDH1 may be used as an additional measure to aid in determining the usefulness of initiating this therapy, understanding its potential risks and benefits.

## Author Contributions

LE and JY conceived and designed the experiments and analyzed and interpreted the data. SK, LE, AL, DB, and MN performed the statistical analysis and bioinformatics. MM, TT, KB, WZ, ML, XF, and B-SL participated in collection and sorting of clinical data. WZ, AL, and MM revised the manuscript critically for important intellectual content.

### Conflict of Interest Statement

The authors declare that the research was conducted in the absence of any commercial or financial relationships that could be construed as a potential conflict of interest.
