# Supplementary material for: ZEB1 Is a Transcription Factor That Is Prognostic and Predictive in Diffuse Gliomas
Source: Front Neurol. 2019 Jan 17;9:1199. doi: 10.3389/fneur.2018.01199 (PMC6345215; doi:10.3389/fneur.2018.01199)
Supplement: Supplementary file 1 [file Data_Sheet_1.docx]

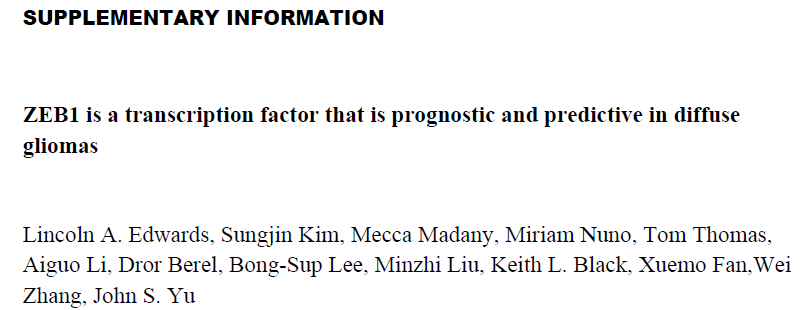


**Supplementary Table I**

**Univariate Association of IDH/ZEB1 with Other Variables in Patients with Grade II/III Gliomas**

| **Variable** | **IDHmut-ZEB1wt (N=257)** | **IDHwt-ZEB1wt (N=14)** | **IDHmut-ZEB1del (N=8)** | **IDHwt-ZEB1del (N=55)** | **P-value** |
| --- | --- | --- | --- | --- | --- |
| Histologic type | | | | | |
| Astrocytoma | 68 (26.46) | 6 (42.86) | 3 (37.5) | 35 (63.64) | <.001 |
| Ambiguous Histology * | 73 (28.4) | 2 (14.29) | 0 (0) | 12 (21.82) |  |
| Oligodendrogliomas | 116 (45.14) | 6 (42.86) | 5 (62.5) | 8 (14.55) |  |
| Grade | | | | | |
| II | 140 (54.47) | 7 (50) | 1 (12.5) | 5 (9.09) | <.001 |
| III | 117 (45.53) | 7 (50) | 7 (87.5) | 50 (90.91) |  |
| 1p/19q co-deletion status | | | | | |
| True | 72 (44.17) | 0 (0) | 4 (80) | 0 (0) | <.001 |
| False | 91 (55.83) | 11 (100) | 1 (20) | 35 (100) |  |
| Age at diagnosis | 39 (33 - 55) | 45.5 (33 - 55) | 43 (33 - 55) | 57 (33 - 55) | <.001 |
| Data are presented as number of patients (column %) or median (IQR, interquartile range). P-value is calculated by Kruskal-Wallis test for age; and chi-square or Fisher’s exact test for categorical variables, where appropriate.  * Formerly Oligoastrocytoma. | | | | | |


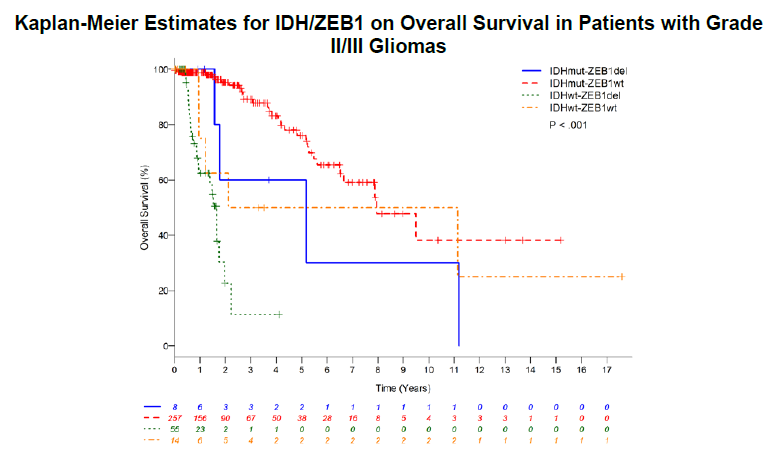


Supplementary Figure 1: Estimated Kaplan-Meier curves for IDH1 mutant or wildtype in conjunction with ZEB1 deletion or wildtype genes in low grade glioma patients
